# Ultrafast soliton and stretched-pulse switchable mode-locked fiber laser with hybrid structure of multimode fiber based saturable absorber

**DOI:** 10.1038/s41598-018-34762-4

**Published:** 2018-11-06

**Authors:** Fengyan Zhao, Yishan Wang, Hushan Wang, Zhijun Yan, Xiaohong Hu, Wei Zhang, Ting Zhang, Kaiming Zhou

**Affiliations:** 1 State Key Laboratory of Transient Optics and Photonics, Xi’an Institute of Optics and Precision Mechanics, Chinese Academy of Sciences, Xi’an, 710119 China; 2 University of Chinese Academy of Sciences, Beijing, 100049 China; 3 Collaborative Innovation Center of Extreme Optics, Shanxi University, Taiyuan, 030006 China; 4 The School of Optical and Electronic Information, National Engineering Laboratory for Next Generation Internet Access System, Huazhong University of Science and Technology, Wuhan, 430074 China

## Abstract

We demonstrate an all-fiber mode locked laser based on hybrid structure of multimode fiber saturable absorber (SA) that can realize both conventional soliton and stretched-pulse states. Stable 16.44 MHz conventional soliton pulses are achieved by injecting 80 mW threshold pump power. By increasing the incident pump power to 420 mW, the laser evolves from soliton operation into stretched-pulse mode locking state. 310 fs stretched-pulse are obtained with the same repetition rate as the soliton pulses. The center wavelength and its 3 dB spectrum bandwidth are 1603 nm and 14.2 nm, respectively. For the first time, we experimentally confirm transition between conventional soliton and stretched-pulse in 1.5 μm mode-locked fiber laser by introducing multimode optical fiber SA. Moreover, the maximum single pulse energy of nearly 1 nJ is achieved. Such all-fiber mode-locked lasers based on hybrid structure of multimode fiber are attractive for practical applications without damage and the limitation of life time.

## Introduction

Ultrafast pulse fiber lasers have attracted extensive attentions due to their merits of compactness, high stability and reliability^1^. In order to achieve picosecond and femtosecond optical pulses, passively mode-locking techniques based on saturable absorber are widely used. As core components, ideal SAs possessing excellent optical properties such as broad operation bandwidth, high optical damage threshold and low saturation intensity are in great demand^2,3^. Presently, the semiconductor saturable absorber mirrors (SESAMs) are widely adopted in mode-locked fiber lasers^4^. However, the application of SESAMs is limited due to their high-cost and limited operation bandwidths. Some two-dimensional (2D) materials based SAs have been proven to exhibit an ultrafast SA property, such as topological isulators (TIs)^5^, carbon nanotubes (CNT)^6^, black phosphorus^7^ and transition metal dichalcogenides (TMDs)^8^. These 2D materials not only possess broad operation wavelength band, but also have unique thickness-dependent bandgap. However, the gradually degraded characteristics and relatively low optical damage threshold of these SAs are the major obstacles in practical application.

To realize ideal SA, multimode fibers (MMFs) have been extensively studied in recent years. MMFs provide additional channels for information transmission and enable high power to transmit through. In the latest literatures, some new nonlinear phenomena and dynamics are observed in MMFs, such as multimode solitons formation^9^, super continuum generation^10^, mode cleaning effect^11^, dispersive waves^12^, and spatiotemporal instability^13^. Besides, some researchers have reported mode-locked fiber lasers using the integration of MMFs^14^. In 2012, Mafi *et al*. reported ultralow-loss couplers where the nonlinear multimode interference (MMI) works^15^. Later, by using graded-index multimode fiber (GIMMF), Hofman *et al*. studied MMI based mode-field adapters in detail^16^. The so-called MMI is that each of excited modes periodically produces interference pattern along it when light transmits in multimode optical fiber. In the linear case, the transmission is a periodic function of GIMMF length. In the nonlinear regime, self-phase and cross-phase modulation effects could lead to the refractive index variation of excited mode when higher power transmits through the GIMMF. Therefore, the generated nonlinear phase delay will alter the interference pattern. In the year of 2013, Nazemosadat *et al*. theoretically researched nonlinear MMI effect and SA behavior by adopting a segment of GIMMF^17^. In theory, the transmission property of the SA depends on the following values: the mode number in GIMMF, the length of GIMMF, the incident pump power, the ratio of optical field diameters between the LG_00_ mode of GIMMF and the fundamental mode of single mode fiber (SMF). Until now, no mode-locked pulses have been experimentally generated based on this SA yet directly. Mafi notes that only when the length of a GIMMF is controlled at an odd number multiplying the beat length, could optical SA be obtained. However, self-imaging beat length of light propagating along a GIMMF is on the order of micrometers, so it is difficult to finely tune the length of a GIMMF in practice. To improve the SA based on MMF, Wang fused a segment of step-index multimode fiber (SIMMF) ahead of GIMMF in experiment^18^. Higher order modes of GIMMF were excited and an effective SA behavior was realized. However, the average output power was only at the level of microwatt. In 2018, Zhao *et al*. optimized the lengths of SIMMF and GIMMF and realized 24 mW average output pulses which results in 2.44 nJ single pulse energy^19^.

Additionally, it is also interesting and important to realize both conventional soliton pulses and stretched pulses mode-locked states in one laser setup. Soliton can be easily taken shape in the negative dispersion regime when anomalous dispersion interacts with and balances the nonlinear Kerr effect in the cavity^5–7^. The merit of soliton pulses is that its shape is easily maintained during transmission, therefore, the information transmitted is more accurate. Thus, these soliton fiber lasers are at the core of optical communication, sensing, optical frequency comb, nonlinear optics and so forth. For stretched-pulse fiber lasers, dispersion plays a critical role when ultrafast pulses propagate within optical fibers. By alternating normal and anomalous group velocity dispersion (GVD) in the cavity, pulses periodically experience stretching and compression. Compared with mode-locked soliton pulses, wider spectrum bandwidths and thus shorter pulse durations can be realized. These stretched-pulse fiber lasers with high output pulse energy have a wide range of applications in the domains of optical amplification, spectroscopy and optical frequency doubling. In 2014, Sotor reported the 128 fs stretched-pulse centered at 1565 nm with the help of TI^20^. Later, they obtained the sub-90 fs stretched-pulse by employing a graphene SA at 1.5 μm^21^. In 2018, Han reported conventional soliton or stretched pulses formation through employing nanotube based SA^22^.

In this paper, we demonstrate a soliton-pulse evolving into stretched-pulse fiber laser by using the hybrid structure of SIMMF-GIMMF as SA for the first time. Contributed by the GIMMF, the laser can work at soliton pulse or stretched-pulse mode locking state via simply changing the pump power. Smooth stretched-pulse spectrum centered at 1603 nm with 3 dB spectral bandwidth up to 14.2 nm is observed. The obtained stretched-pulse with 310 fs pulse duration is, to the best of our knowledge, the shortest pulse generated from 1.5 μm waveband oscillators mode locked by the SA so far. The output single pulse energy can reach almost 1 nJ-level. The SIMMF-GIMMF SA exhibits high pump power tolerance and great potential for high power, large energy fiber laser generation. It is attractive to operate this high energy ultrafast soliton and stretched-pulse switchable mode locking fiber laser for a range of applications in practice.

## Results

### Preparation and characterization of SIMMF-GIMMF based SA

**Figure 1:**
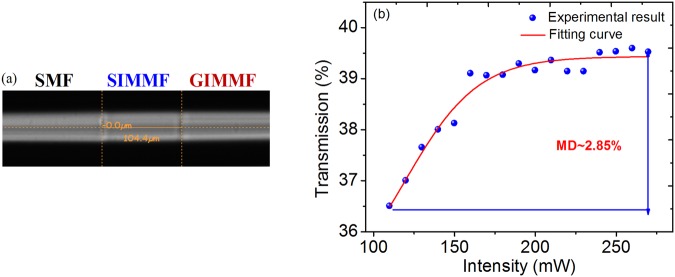
The microscopy image and nonlinear transmission property of the SIMMF-GIMMF SA. (**a**) The microscopy image. (**b**) Nonlinear transmission property.

In this experiment, a segment of 104.4 μm SIMMF (105/125) is connected with 20 cm long GIMMF (50/125). Figure 1(a) displays the microscopy image of SIMMF-GIMMF SA. By bending the section of SA, the mode field distribution is dramatically affected, which in turn results in the variation of the MMI. Through this method, the ratio of LG_00_ mode-field diameters coupling to GIMMF and the excited ones is adjustable. Therefore, the restriction on the length of the GIMMF can be eliminated. We test different lengths of SIMMF from 100 μm to 500 μm for the SA and find that it is a good strategy to get higher output power by reducing the length of SIMMF. Therefore, about 100 μm length of the SIMMF which almost reaches the limit on fiber splice is chosen in this experiment. The nonlinear transmission property is measured by using a standard 2-arm measurement system. The light source is a home-made mode-locked fiber laser (1560 nm central wavelength, 4 nm bandwidth within 3 dB, 10 MHz pulse repetition rate). From Fig. 1(b), the SIMMF-GIMMF structure exhibits typical characteristics of a SA. Explicitly, a modulation depth of 2.85% is smaller than that of MoS_2_^23^, resulting in a lower mode locking threshold for laser oscillators.

### Mode locked fiber laser with SIMMF-GIMMF based SA

**Figure 2:**
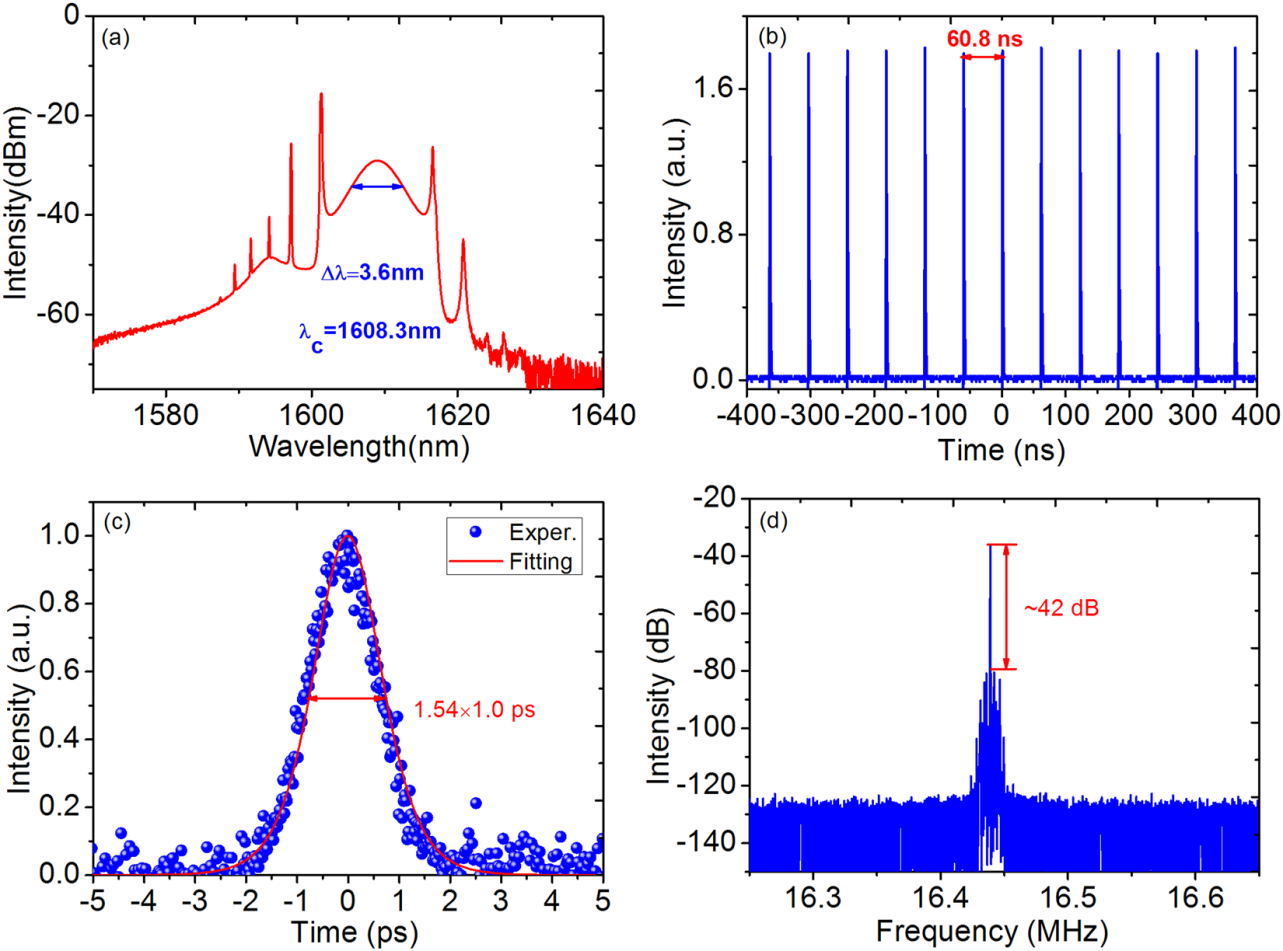
Soliton pulse operation based on SIMMF-GIMMF SA. (**a**) Recorded conventional soliton spectrum centerd at 1608.3 nm. (**b**) Single-pulse train. (**c**) Autocorrelation trace obtained by 10% output ratio. (**d**) Measured RF spectrum.

At first, by properly adjusting two PCs and curves of SA, self-started conventional soliton mode-locking with 16.44 MHz repetition rate can be initiated at only 80 mW pump power. Figure 2 presents the measured properties of soliton operation at 150 mW injected pump power. The center wavelength is 1608.3 nm and 3 dB spectral bandwidth is around 3.6 nm, as shown in Fig. 2(a). The output spectrum with symmetrical located sidebands presents an obvious characteristic for conventional soliton mode locking operation. Figure 2(b) exhibits a stable and uniform pulse train with an interval of 60.8 ns. Meanwhile, as exhibited in Fig. 2(c), the mode-locked pulse width assuming Sech^2^ fitting curve is 1.0 ps. The time bandwidth product (TBP) is calculated to be 0.416, indicating that the obtained conventional soliton pulse almost reaches transform limited state. In Fig. 2(d), the radio-frequency (RF) spectrum with signal-to-noise ratio (SNR) of 42 dB is recorded under 10 Hz resolution and 400 kHz span, suggesting high mode-locking stability.

**Figure 3:**
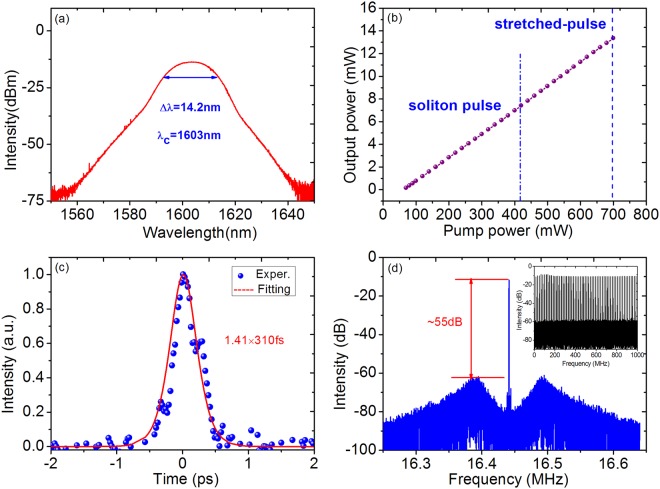
Stretched-pulse operation characteristics at 16.44 MHz frequency repetition rate. (**a**) Spectrum centered at 1603 nm. (**b**) The linear variation of average output power versus injected pump power. (**c**) Autocorrelation trace obtained by 10% output ratio. (**d**) Measured RF spectrum profile. Inset: the wide span RF spectrum.

Stable soliton pulses could be kept if the injected pump power is below 390 mW. By further slightly increasing the pump power with PCs unchanged, we observe that the mode locking state becomes unstable and the fluctuation waveform of single-pulse train is obvious. This kind of multi-pulsing instability attributes to excessive non-linear phase induced by GIMMF in the mode-locked laser cavity^14^. Once the incident power exceeds 420 mW, stretched-pulse operation can be achieved instead of conventional soliton state. As shown in Fig. 3(a), smooth stretched-pulse spectrum centering at 1603 nm with 3 dB spectral bandwidth up to 14.2 nm is observed. As the pump power is raised to 700 mW, stable stretched pulse mode locking state could still be maintained. Moreover, the spectrum bandwidth keeps unchanged in the whole stretched process. The largest output average power is 14 mW, suggesting that intracavity pulse energy is 0.85 nJ, which is lower than our previously reported result due to the shorter length of GIMMF^19^. Figure 3(b) displays the variation of average output power from the 10% output port with the injected pump power. Obviously, the average output power is almost linear function as the incident power with calculated slope efficiency of 31%. Interestingly, the average output power in experiment has no obvious changes when the mode-locking state is switched from soliton to stretched-pulse. It is deduced that, when no pulse breaking exists in the laser, the pump and loss in the cavity rather than the mode-locking state are mainly responsible for the output power. This phenomenon will be further studied and confirmed in future works. Figure 3(c) presents the measured autocorrelation trace which indicates the stretched-pulse width of 310 fs through Gaussian fitting curve. Therefore, the TBP of the output stretched-pulse is 0.513, implying that the output pulses are slightly chirped. Furthermore, the fundamental repetition rate of the stretched-pulse is the same as that of soliton, indicating that the optical path remains unchanged in the whole experiment. In addition, the SNR is measured to be around 55 dB with the same resolution bandwidth and span as that of the conventional soliton RF spectrum, as illustrated in Fig. 3(d), and the inset shows the wide span RF spectrum clarifying stable stretched-pulse mode locking operation. As long as the experimental conditions are kept unaltered, the stretched-pulse fiber laser is able to work stably for several days continuously.

**Figure 4:**
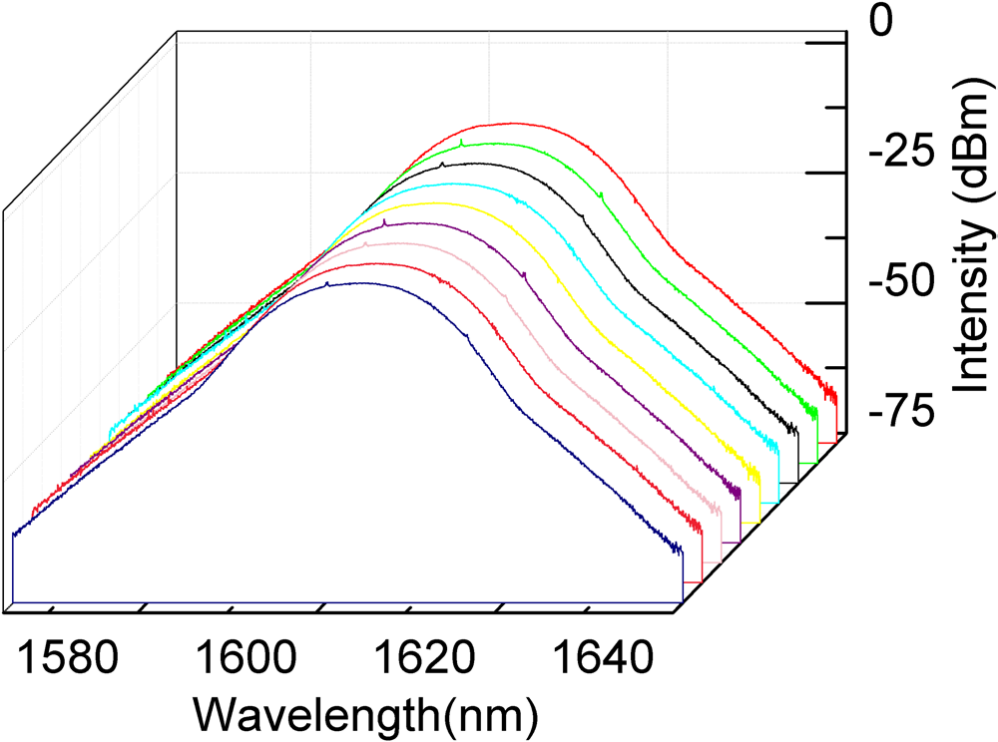
Spectrum stability of stretched-pulse mode-locking operation. Spectra recorded at an interval of 40 minutes for 6 hours.

To evaluate stability of the oscillator in long terms, the stretched-pulse spectra are monitored and recorded every 40 minutes for total measuring time of 6 hours, as depicted in Fig. 4. Evidently, the spectra stay almost constant indicating excellent long time stability.

**Figure 5:**
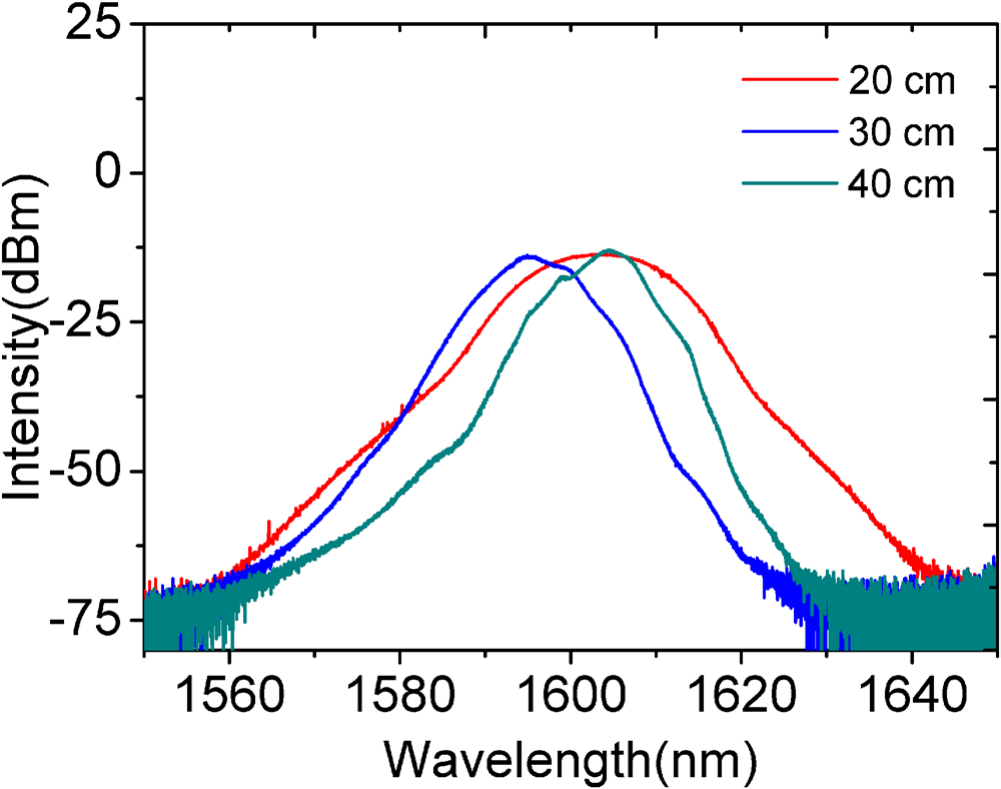
Spectra of stretched-pulse under different lengths of GIMMF at pump power of 700 mW.

The measured optical spectra of the stretched-pulse with different length of GIMMF are sketched in Fig. 5. As illustrated in the figure, the spectral bandwidth can vary dramatically under different GIMMF length which attributes to diverse phase delay values of higher order modes in GIMMF at higher pump power. Meanwhile, the change of the center wavelength mainly attributes to the varying of the PCs state and different SA curvatures.

Additionally, a polarization-independent isolator is chosen without initiating nonlinear polarization rotation in this experiment. To further verify the contribution of the SIMMF-GIMMF SA to mode-locking operation, the SA section is removed from the fiber laser. On this condition, no mode-locked pulses can be observed in despite of carefully adjusting the intracavity polarization state and pump power.

## Discussion

The transition between conventional soliton and stretched-pulse based on SIMMF-GIMMF SA could mainly attribute to two factors including the dispersion and nonlinear effects of the GIMMF. The net intracavity dispersion of soliton mode-locked state is calculated by using the location of spectral sidebands given by Eq. (1)^24^

1 $${\rm{\Delta }}{\lambda }_{N}=\pm \sqrt{\frac{2N}{cDL}-0.00787\frac{{\lambda }_{0}^{2}}{{(c\tau )}^{2}}}$$

Where N, *λ*_0_, *τ*, D are resonance series, central wavelength, pulse width of soliton, dispersion parameter, respectively. Besides, DL is related to the net intracavity dispersion *β*_2_L through Eq. (2).

2 $${\beta }_{2}L=-\,\frac{DL\cdot {\lambda }_{0}^{2}}{2\pi c}$$

The result of (2) is calculated to be −0.443 ps^2^, obviously smaller than the experimentally estimated value of −0.056 ps^2^ due to the complicated dispersion property of GIMMF. With the increase of pump power, the dispersion of higher-order modes and inter-modal dispersion in the GIMMF could be altered. It is estimated that when the pump power reaches to some level in this experiment, the dispersion of GIMMF leads to a total intracavity dispersion which is more suitable for stretched-pulse mode locking operation. From the perspective of nonlinear effects, the spectral bandwidth will change with the variation of the phase delay. The bandwidth of the PC and MMF-induced spectral filter is given by Δ*λ* = 2*πλ*/Δ*ϕ* ^25^, where *λ* is the wavelength, and *ϕ* is the phase shift accumulated through MMF. By using large core MMF, the non-linear phase-shift in the cavity is reduced for a certain peak power^18^. Therefore, the bandwidth could be increased, which helps to the evolution between soliton and stretched-pulse. Thanks to the MMF, it provides the bridge between conventional soliton-pulse and stretched-pulse in all-fiber lasers.

## Methods

**Figure 6:**
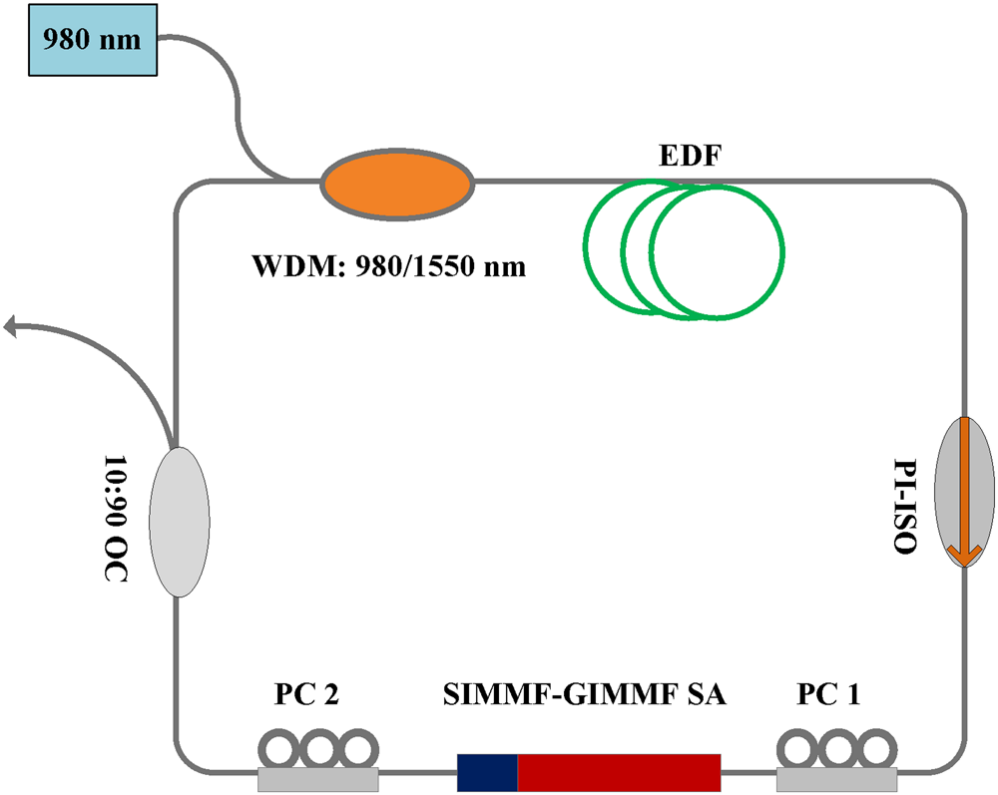
Schematic of the oscillator with SIMMF-GIMMF SA.

The schematic of the oscillator based on SIMMF-GIMMF SA is sketched, as shown in Fig. 6. A section of the SIMMF-GIMMF is spliced in the cavity to realize effective SA function. The cavtiy contains a 2.8 m long highly erbium-doped gain fiber (EDF, Nufern SM-ESF-7/125) which is pumped through a 980 nm diode laser source (700 mW maximum output power), a wavelength-division multiplexer (WDM) coupler transmission at 980/1550 nm, a polarization-insensitive isolator (PI-ISO), two sets of polarization controllers (PCs) and an optical coupler (OC) with 10% output port utilized to monitor the laser output characteristics. Besides the gain fiber, the remaining fibers are standard SMFs. The total ring cavity length is around 12.6 m. The group dispersion at 1.56 μm for EDF and SMF are 59.74 ps^2^/km and −23.25 ps^2^/km, respectively. Therefore, the total dispersion value is about −0.056 ps^2^, which facilitates the formation of conventional soliton. The output pulses are monitored via a series of devices including an optical spectrum analyzer (Yokogawa AQ6370D, 0.02 nm spectral resolution), a digital oscilloscope (Tektronix TDS784D), a commercial autocorrelator (AInair HAC200) and a RF spectrum analyzer (Agilent technologies N9000A).

## Conclusion

In conclusion, we produce a hybrid structure of SIMMF-GIMMF SA with 2.85% modulation depth in our experiment. Base on this SA, a soliton and stretched-pulse switchable mode locking fiber laser is demonstrated. By raising injected pump power to 80 mW, soliton pulses with 1.0 ps pulse width are obtained at 16.44 MHz fundamental repetition rate. Conventional soliton pulses can automatically switches to stretched-pulse mode-locked state as long as injected pump power exceeds 420 mW. In this situation, the laser delivers 310 fs stretched pulses. The spectrum centering at 1603 nm with 3 dB spectral bandwidth up to 14.2 nm is achieved. For the first time, we experimentally confirm transition between conventional soliton and stretched-pulse based on the application of the hybrid structure of multimode optical fiber as SA in 1.5 μm mode-locked fiber laser. Moreover, 310 fs is the minimum pulse duration obtained based on the SA in erbium–doped fiber lasers. Besides, nearly 1 nJ single pulse energy is obtained in our experiment. These results experimentally indicate that SIMMF-GIMMF SA is feasible to form high energy femtosecond pulses.
